# Commentary: A rare case report of *Salmonella* infection: severe necrotizing pneumonia with empyema in an immunocompetent child

**DOI:** 10.3389/fped.2026.1834587

**Published:** 2026-05-04

**Authors:** Regina Célia de Souza Campos Fernandes, Enrique Medina-Acosta

**Affiliations:** 1 Faculdade de Medicina de Campos, Campos dos Goytacazes, Brazil; 2 Hospital Plantadores de Cana, Campos dos Goytacazes, Brazil; 3 Laboratório de Biotecnologia, Universidade Estadual do Norte Fluminense, Campos dos Goytacazes, Brazil

**Keywords:** clinical reasoning, immunocompetence, invasive bacterial infection, necrotizing pneumonia, non-typhoidal Salmonella, sentinel clinical events

## Introduction

The recent case report by Sabbahi and colleagues (1) describes an unusual and severe presentation of invasive non-typhoidal *Salmonella* infection in a previously healthy four-year-old child who developed necrotizing pneumonia with empyema requiring extracorporeal membrane oxygenation. Pulmonary infection caused by non-typhoidal *Salmonella* is exceedingly rare, particularly among immunocompetent pediatric patients, as these organisms most commonly cause self-limited gastrointestinal disease (2). The report therefore raises an immediate clinical question: how can a pathogen that typically causes self-limited gastrointestinal illness produce life-threatening pulmonary disease in an otherwise healthy child?.

When a pathogen that typically produces mild gastrointestinal illness causes life-threatening invasive disease in an otherwise healthy child, clinicians are naturally prompted to reconsider the factors that may have contributed to such an aggressive presentation, particularly given that invasive non-typhoidal *Salmonella* infections are associated with substantial morbidity and mortality worldwide (2). Addressing this question does not necessarily require identifying a single causal mechanism; rather, it invites reflection on how unusual infections may reveal important insights into host–pathogen interactions and clinical reasoning in pediatric practice.

## The clinical paradox of invasive *Salmonella* infection in an immunocompetent child

One of the most striking features of the case described in the report (1) is the discrepancy between the typical clinical behavior of the pathogen and the severity of the disease observed. Non-typhoidal *Salmonella* infections are generally associated with self-limited gastroenteritis, while extraintestinal manifestations are relatively uncommon, particularly in children without underlying medical conditions (2). The development of necrotizing pneumonia with empyema in a previously healthy child therefore represents a notable clinical paradox.

In everyday clinical practice, pediatricians often rely on pattern recognition when evaluating infectious diseases. Each pathogen is associated with a spectrum of expected clinical presentations. When an organism appears in an unexpected anatomical site or produces unusually severe pathology, clinicians are naturally prompted to reconsider the factors that may have contributed to that outcome.

Several explanations may account for such discrepancies. In some cases, severe infections may arise from stochastic events or transient predisposing conditions. In the reported case (1), the presence of influenza A infection may have facilitated bacterial invasion of the respiratory tract and contributed to disease progression. Viral respiratory infections are well known to predispose patients to secondary bacterial pneumonia, although *Salmonella* remains an exceptionally uncommon pathogen in this setting. Viral respiratory pathogens, such as influenza A, can induce direct cytopathic damage to the respiratory epithelium, impair mucociliary clearance, and trigger viral-induced immune modulation—such as the depletion or desensitization of alveolar macrophages. These mechanisms act synergistically to compromise local host defenses and create a permissive environment for subsequent bacterial adherence and invasion (3).

Alternatively, unusually severe infections may occasionally represent the first clinical manifestation of a previously unrecognized host vulnerability (4). Host defense against intracellular bacteria involves a network of immune mechanisms, including innate pathogen recognition pathways, phagocyte antimicrobial activity, and cytokine-mediated activation of macrophages. Defects affecting any of these systems may remain clinically silent until a specific infectious challenge reveals the underlying vulnerability. In the specific context of invasive non-typhoidal *Salmonella* infections, the interleukin-12 (IL-12) and interferon-gamma (IFN-*γ*) axis plays a critical protective role in intramacrophage bacterial clearance. Deficiencies or subtle genetic variants within the IL-12/IFN-*γ* pathway are extensively recognized as responsible for severe, disseminated *Salmonella* infections in individuals who appear otherwise phenotypically immunocompetent (4).

It is also possible that severe disease arises from complex interactions between host immunity and pathogen virulence rather than from a single identifiable abnormality (5). In the reported case, the patient's deterioration was accompanied by laboratory findings suggestive of a hyperinflammatory response, raising the possibility that host inflammatory amplification may have contributed to pulmonary tissue damage and disease severity.

## When unusual infections function as sentinel clinical signals

Cases such as the one described by Sabbahi and colleagues (1) illustrate an important principle in clinical medicine: unusual infections can function as diagnostic signals embedded within routine practice. When a pathogen that typically causes mild disease produces a severe or atypical manifestation, clinicians are prompted to reassess whether the event reflects an exceptional infection, an unrecognized host susceptibility, or a combination of both.

In this sense, the present case may be interpreted as a sentinel clinical event—an occurrence that signals the need to reconsider host–pathogen dynamics when disease severity exceeds typical expectations.

The designation of a patient as “immunocompetent” in clinical practice generally reflects the absence of recognized immunodeficiency and normal findings in initial laboratory screening. However, immune defense against infection is complex and involves multiple interacting biological pathways. Subtle vulnerabilities in these systems may remain undetected during routine clinical evaluation yet become apparent when a pathogen challenges a particular immune mechanism. The types of diagnostic questions that such sentinel cases may raise in clinical practice are summarized in Table 1.

**Table 1:** Clinical reasoning triggered by unusually severe or atypical infections in apparently immunocompetent children.

Clinical observation	Diagnostic question raised	Possible underlying mechanisms	Practical clinical considerations
Severe extraintestinal infection caused by a pathogen typically associated with mild disease	Why did the infection evolve with such severity?	Host–pathogen interaction imbalance; unusually high inoculum; pathogen virulence factors	Reassess the clinical context and potential contributing factors
Unusual anatomical localization (e.g., pulmonary *Salmonella* infection)	Why is the pathogen present in this anatomical site?	Transient tissue vulnerability (e.g., antecedent viral respiratory infection), local epithelial damage, impaired mucociliary clearance	Investigate preceding infections or structural lung conditions
Severe infection in a previously healthy child	Could this represent the first manifestation of host susceptibility?	Subtle immune defects affecting intracellular bacterial control, phagocyte function, or innate immune sensing	Consider immunological evaluation if clinical suspicion persists
Rapid clinical deterioration despite appropriate antimicrobial therapy	Could host inflammatory responses contribute to disease severity?	Hyperinflammatory responses, cytokine-mediated tissue injury	Monitor inflammatory markers and consider immune-mediated complications
Absence of identifiable risk factors for invasive infection	Is the classification “immunocompetent” sufficient?	Undetected or pathway-specific immune vulnerabilities (e.g., localized defects in the IL-12/IFN-*γ* axis)	Consider referral for specialized immunological assessment if clinically indicated

Recognizing unusual infections as sentinel signals may therefore encourage clinicians to consider broader diagnostic perspectives. Such cases remind practitioners that rare clinical presentations can provide valuable insight into the limits of host immune defenses and into the diversity of host–pathogen interactions observed in pediatric infectious disease.

## Discussion

The case reported by Sabbahi and colleagues (1) contributes to the literature by documenting a rare manifestation of pulmonary *Salmonella* infection in a previously healthy child. Beyond its rarity, however, the report highlights the importance of clinical reasoning when confronted with infections that appear disproportionate to the expected pathogenic behavior of the organism.

Rather than serving solely as a description of an uncommon infection, the case invites reflection on the broader biological and clinical questions that such presentations raise. When clinicians encounter unusually severe infections caused by pathogens typically associated with mild disease, particularly in the context of invasive non-typhoidal *Salmonella* infection, which is associated with substantial morbidity and mortality worldwide (6, 7), it becomes important to consider whether the event reflects stochastic infection, transient host susceptibility, or a more subtle vulnerability in immune defense mechanisms.

By emphasizing this diagnostic perspective, the report encourages pediatricians, infectious disease specialists, and immunologists to remain attentive to the clinical signals embedded within unusual infections.

Rare cases not only expand the recognized spectrum of disease but also serve as entry points for understanding the biological mechanisms that shape host susceptibility to infection, particularly in the context of invasive non-typhoidal *Salmonella* disease, which remains a significant cause of severe infection globally (2). Recognizing and interpreting these signals within routine clinical practice remains an essential component of pediatric infectious disease and clinical immunology.
